# Pathogenesis and Disease Control in Crops: The Key to Global Food Security

**DOI:** 10.3390/plants12183266

**Published:** 2023-09-14

**Authors:** Temoor Ahmed, Muhammad Noman, Muhammad Shahid, Amir Hameed, Bin Li

**Affiliations:** 1 Xianghu Laboratory, Hangzhou 311231, China; temoorahmed@zju.edu.cn; 2 Institute of Biotechnology, Zhejiang University, Hangzhou 310058, China; m.noman@zju.edu.cn; 3 State Key Laboratory for Managing Biotic and Chemical Threats to the Quality and Safety of Agro-Products, Key Laboratory of Biotechnology in Plant Protection of Ministry of Agriculture and Rural Affairs and Zhejiang Province, Institute of Plant Protection and Microbiology, Zhejiang Academy of Agricultural Sciences, Hangzhou 310021, China; 4 Department of Bioinformatics and Biotechnology, Government College University, Faisalabad 38000, Pakistan; mshahid@gcuf.edu.pk; 5 Plant Breeding and Acclimitization Institiue—IHAR, 05-870 Radzikow, Poland; a.hameed@ihar.edu.pl

Plant diseases are a major threat to global food security. They can cause significant losses in crop yields, and can also lead to the spread of harmful toxins [1]. The pathogenesis of plant diseases is a complex process that involves the interplay between pathogens, hosts, and their environment [2,3]. The ever-evolving nature of pathogens and the effects of climate change on plant health make it essential to develop effective disease control strategies. A multi-pronged approach to disease control is imperative. Integrated pest management (IPM) strategies that incorporate cultural practices, biological controls, and targeted chemical interventions can help to minimize environmental impact while effectively tackling crop diseases [4]. Breeding disease-resistant cultivars through modern biotechnological tools is another avenue for curtailing pathogenesis and reducing reliance on chemical treatments [5]. Global collaboration is also essential for sharing insights, data, and successful strategies to help vulnerable regions fortify their defenses against emerging threats. Recent research plays a pivotal role in disseminating best practices among farmers, enabling them to make informed decisions and implement preventive measures [6,7]. By investing in research, technology, and international cooperation, humanity can cultivate resilience in agriculture and surmount the challenges posed by plant diseases [8,9].

This Special Issue of the journal “Plants” covers a range of topics related to the latest research trends in pathogenesis and disease control in crops for sustainable agriculture. The twelve original research articles and one review article in this Special Issue contribute to what we know about disease control in crops by providing new insights. These contributions shed light on the molecular mechanisms of pathogenesis, the development of new disease control strategies, the use of biotechnology to improve disease resistance, and the impact of climate change on plant diseases. For example, Ibrahim et al. [10] revealed the potential of endophytic bacteria, and argued that the *Pseudomonas poae* strain CO can enhance wheat growth and can help to combat Fusarium seedling blight disease. The strain’s multifaceted antifungal properties and growth-promoting attributes make it a promising alternative to synthetic chemicals for safeguarding wheat against fungal infections. In another study, Kawaguchi et al. [11] developed a new pathogen–healthy–latently–infectious–diseased (PHLID) model for tomato bacterial canker (TBC) disease, offering insights into disease dynamics and control strategies. It successfully captured disease incidence and control effects, providing a valuable tool for simulating and managing TBC outbreaks. Likewise, Haq et al. [12] developed integrative pathogenicity (IP) postulates for authenticated pathogenicity testing in plants. The IP postulates are based on the identification of effector genes associated with host pathogenicity, which are determinant markers for virulence. The IP postulates were validated by confirming the identity of a new forma specialis of *Fusarium oxysporum* that causes vascular wilt in datepalm. 

Some studies also reported the application of nanomaterials for plant disease management. For example, silver nanoparticles (AgNPs) in combination with *Bacillus amyloliquefaciens* CFCS present a promising approach to help mitigate sweet potato soft rot disease, curbing pathogen growth while minimizing environmental nanoparticle contamination [13]. In another study, biogenically synthesized AgNPs from *Arctium lappa* fruit, *Solanum melongena* leaves, and *Taraxacum mongolicum* leaves hold promising antibacterial potential against rice bacterial leaf blight, showcasing the effective inhibition and disruption of *Xanthomonas oryzae* pv. *oryzae* [14]. Similarly, Francesconi et al. [15] introduced an innovative nanostructured particle formulation (NPF) as an eco-sustainable solution for managing *Fusarium* head blight and *Fusarium* crown rot in compliance with the evolving environmental regulations, offering promising control and elicitor effects. Another study investigated the bacterial blight disease of cotton caused by *Xanthomonas citri* subsp. *malvacearum* (Xcm). The study found that the scratch method was the most effective for perpetuating Xcm in cotton varieties. The aqueous plant extracts of *Aloe vera*, *Mentha piperita*, *Syzygium cumini*, and *Azadirachta indica* showed the most promising antibacterial activity against Xcm pathogen [16]. In another study, Pérez-González et al. [17] found that wheat bran and amaranth are the best protein sources for stimulating the growth and conidiation of the entomopathogenic fungus *Hirsutella citriformis*. The gum produced by *H. citriformis* can be used to formulate conidia, which can improve the biological control of *Diaphorina citri* adults. Li et al. [18] demonstrated that bayberry decline disease is associated with soil acidity and aluminum toxicity. The results suggest that mitigating the disease may be possible by regulating soil parameters, such as pH and aluminum concentration. On the other hand, a study found that tomato brown rugose fruit virus (ToBRFV) is a serious threat to tomato production in Saudi Arabia. The virus is highly virulent and can infect a wide range of tomato cultivars [19]. The review by Lv et al. [20] reported the beneficial and harmful effects of *Pantoea* bacteria on rice growth. The beneficial effects include growth promotion, abiotic alleviation, and disease inhibition, while the harmful effects include virulence and pathogenicity. The study also discussed the existing scientific problems and future research prospects in this field. In summary, the articles in this Special Issue are of interest to researchers, students, and other professionals working in the fields of plant pathology, crop science, and sustainable agriculture. They provide a valuable resource for anyone who is interested in learning more about the latest advances in disease control in crops.
